# Evaluation of MMR live attenuated vaccine oncolytic potential using Ehrlich ascites carcinoma in a murine model

**DOI:** 10.1007/s12032-022-01866-x

**Published:** 2022-10-29

**Authors:** Sara T. Hassan, Aly F. Mohamed, Nourhan H. AbdelAllah, Hamdallah Zedan

**Affiliations:** 1 Laboratory Evaluation Administration, Egyptian Drug Authority, Giza, 12654 Egypt; 2 International Center for Training and Advanced Researches (ICTAR-Egypt), Cairo, Egypt; 3 Department of Microbiology and Immunology, Faculty of Pharmacy, Cairo University, Cairo, 11562 Egypt

**Keywords:** Oncolytic virus, Tumor, MMR, Vaccine, Ehrlich ascites carcinoma

## Abstract

MMR vaccine is a common vaccine that contains oncolytic viruses (Measles, Mumps, and Rubella) and could be used as a potential anti-cancer treatment. In this study, we assessed the anti-tumor activity of the MMR vaccine against Ehrlich ascites carcinoma (EAC) solid tumor induced in mice. The in vitro assay showed that vaccine IC50 in EAC was approximately 200 CCID_50._ The vaccine was intratumorally administrated twice weekly in EAC-bearing mice. The antitumor response of the vaccine was measured by tumor growth, survival rate, histopathologic examination, flow cytometry analysis, and body biochemical parameters. The MMR vaccine demonstrated a substantial reduction of tumor growth and prolongation of life span as well. The proliferation marker was significantly lower in the vaccine-treated group. Moreover, the apoptosis key parameter Casp-3 was also higher in the vaccine-treated group. The vaccine somewhat restored the deterioration of the biochemical parameters (LDH, GOT, GPT, MDA, NO, and PON-1) in the tumor-bearing mice. Finally, this study indicated the potential antitumor effect of MMR vaccine via anti‑proliferative, apoptotic activities, and modulating the antioxidant parameters. This study opens a new field of inquiry for future research on the vaccine’s anti-cancer properties.

## Introduction

Cancer is considered one of the leading causes of death, which can affect any part of the body whether an organ or tissue. It occurs when the cells escape apoptosis and loss control of the division. Cancer may occur in one area of the body and spread to other parts of the body which is called metastasis, and it is a leading cause of cancer-related death [1]. Globally, the cancer burden is increasing. Many cancer patients around the world lack the accessibility to high-quality diagnosis and treatment on time, especially in low- and middle-income countries where their health systems are not usually prepared to handle this burden.

Cancer treatments depend on different factors including the type and stage of cancer, which usually are a combination of treatments. The treatments may be local such as surgery or radiation, to treat a specific tumor or body area. While, systemic treatment such as chemotherapy, stem cell, and bone marrow transplant, hormone therapy, and immunotherapy affects the whole body [2, 3].

One of the uprising treatments for cancer is Oncolytic viruses (OVs). OVs are non-pathogenic viral strains that selectively kill cancer cells. They are either genetically engineered or naturally occurring viruses that can replicate in, spread within the tumor and cause lysis of cancerous cells, and also can induce immune responses to the infected tumor cells. Oncolytic virotherapy has many advantages over the traditional treatments including replication of the virus into the tumor leaving normal tissues without damage, a lytic effect resulting in a pro-inflammatory microenvironment and leads to anticancer response [4], virus concentration increases with time, unlike the conventional treatments which decrease with time and low incidence of resistance as viruses target multiple means for cytotoxicity and oncogenic pathways [5]. The mechanism of action of Oncolytic viruses depends on two main mechanisms: direct lysis of cancer cells and induction of antitumor immunity [6–8]. Many virus candidates were investigated as potential oncolytic virotherapy, including adenoviruses, herpes simplex virus (HSV), measles virus (MV), mumps virus (MuV), Newcastle disease virus (NDV), polioviruses, reoviruses, vesicular stomatitis virus (VSV), even Zika virus [9–11]. Many of these viruses have already been used in vaccines that have been licensed around the globe.

Ehrlich ascites carcinoma (EAC), a spontaneous adenocarcinoma in mice, is a well-known tumor model that has been widely employed in the research of tumor pathogenesis and the development of anti-tumorigenic drugs. It is an undifferentiated carcinoma with high transplantation capacity, high proliferation rate, and shorter life span [12, 13].

Measles, Mumps, and Rubella (MMR) vaccine is one of the live attenuated vaccines, it is a safe and effective combined vaccine that has been widely employed. The goal of this study is to evaluate the oncolytic effects of licensed combined (MMR) live attenuated vaccine using Ehrlich ascites carcinoma (EAC) in a murine model in vitro and in vivo.

## Materials and methods

### Animals and vaccine

Female Swiss albino mice weighing about 20 ± 2 g were used. Mice were housed in polyacrylic cages and kept under standard laboratory conditions (temp. 25 ± 2 °C, humidity 50 ± 5%, and 12/12 h light/dark cycle), they were fed with a standard diet and water freely. Mice were kept at least 1 week before starting the experiment for acclimatization.

MMR vaccine (Tresivac®) from Serum Institute of India (SII) was used containing live attenuated measles virus (Edmonston-Zagreb MV strain) with not less than 1000 CCID50 of MV, mumps virus (L-Zagreb MuV strain) with not less than 5000 CCID50 of MuV, and rubella virus RV (Wistar RA 27/3 RV strain) with not less than 1000 CCID50 of RV in each human dose of 0.5 ml.

### Tumor cells and tumor induction in mice

Ehrlich ascites carcinoma (EAC) cells were obtained from National Cancer Institute, Cairo University, Egypt. EAC was maintained in mice by intraperitoneal (i.p) injection. The ascitic fluid was collected after 7–10 days from tumor-bearing mice under aseptic conditions. Aspirated EAC cells were tested for viability, counted, and adjusted with sterile isotonic saline to 1 × 10^7^/ml [14]. The solid tumor was induced by inoculation of EAC cells subcutaneously (s.c) with 0.25 ml (2.5 × 10^6^ EAC cells) into the mice [15, 16].

### Cytotoxicity test

EAC cells were dispensed at 1 × 10^5^cells/well in a sterile 96 well plate in Minimum Essential Medium (MEM) (Biowest, France) with 5% fetal bovine serum (Sigma, USA) and left overnight. MMR vaccine was twofold serially diluted and dispensed in triplicates. One column of cells was inoculated with MEM only as negative control wells. The plate was incubated for 48 h at 37 °C and 5% CO_2_. The plate was cold centrifuged (Jouan-Ki22, France), the treatment medium was aspirated and cells were washed to remove phenol red-containing medium residues. Thereafter, 3-[4,5-dimethylthiazol-2-yl]-2, diphenyltetrazolium bromide (MTT) (Sigma-Aldrich, USA) at 0.05 mg/ml was added to the cells/well for 4 h. Later, the MTT was aspirated from the wells and DMSO (Sigma-Aldrich, USA) was added to dissolve the developed formazan crystals. The absorbance was measured at 570/630 nm using a microplate reader (ELX-800 Biotek, USA) and the IC_50_ was calculated using Masterplex 2010 software.

### Experimental design

Thirty mice were injected with EAC cells (2.5 × 10^6^) subcutaneously except the control group (*n* = 6) injected with saline, the injected mice were housed for 14 days for solid tumor formation. Mice were randomly divided into two groups (*n* = 12) and treated as follows:

Group 1: EAC positive treated with saline;

Group 2: EAC positive treated with MMR vaccine.

All test groups were injected at day 14 after tumor inoculation with normal saline or MMR vaccine with 200 µl intratumorally twice per week for 3 weeks. Mice from each group were observed where tumor growth and survival rate were recorded. At the end of the experiment, blood samples were withdrawn for further analysis. Mice have been sacrificed afterward, the tumor mass, liver, and kidney were removed, rinsed in saline, and parts of them were fixed in 10% neutral buffered formalin for further analysis.

### Tumor mass size

The tumor mass was measured and recorded regularly using a digital caliper at weekly intervals (0, 7, 14, and 21 days) post-tumor induction.

### Serum and tissue for biochemical analyses

Sera of collected blood from all mice groups were collected by cold centrifugation for 10 min at 4000 rpm and pooled. After decapitation, the liver and kidney from each mouse were removed, rinsed with ice-cold saline, homogenized then centrifuged at 4000 rpm for 15 min at 4 °C, and the supernatant was obtained for biochemical analysis. The levels of glutamate oxaloacetate transaminase (GOT), glutamate pyruvate transaminase (GPT), and lactic dehydrogenase (LDH) were measured in test sera samples. The content of reduced glutathione (GSH), malondialdehyde (MDA), nitric oxide (NO), and paraoxonase-1 (PON-1) in the tissues of dissected organs was determined as oxidative stress and anti-oxidant factors. GOT, GPT, LDH, GSH, MDA, NO, and PON were analyzed by commercial enzymatic and colorimetric kits using spectrophotometry as described before [17–21].

### Histopathological and immuno-histochemistry examination

Tumor masses prepared slides were stained with Hematoxylin and Eosin (H/E) and examined with a light microscope [22]. The H/E-stained slides were examined and visualized under a light microscope (magnification ×100) (Zeiss, Germany), and photos were captured using a digital image capture system (Olympus BX41, camera model number Olympus DP20 at 40X, USA). Similarly, immune-histochemical staining was used for proliferating cell nuclear antigen (PCNA) on formalin-fixed paraffin-embedded tissues. Staining was performed using the Histostain-Plus kit (Dako, USA) which contains 10% non-immune serum, biotinylated secondary antibody, and streptavidin-peroxidase as previously described [22, 23].

### Flow cytometry analysis

Tumor tissues were excised from EAC-bearing mice, chopped, and gently rubbed through fine nylon gauze (40–50 mesh count/cm; HD 140 Zuricher Buteltuch Fabrik AG, Zürich, Switzerland). Samples were washed through the gauze with Tris–EDTA buffer, pH 7.5. Cells were suspended in PBS, centrifuged for 5 min at 200–300 g, resuspended in sterile PBS and cell density adjusted to 1 × 10^6^ cells/ml, and fixed in 70% ice-cold methanol in PBS and stored at − 20 °C until used. The apoptosis profile was determined in tumor tissue culture cells using flow cytometry by a commercial Annexin V-FITC flow cytometry Kit (Abcam, USA).

### Western blotting analysis

Caspase-3 (Casp-3) protein was analyzed in tumor specimens by Western Blotting [18]. In brief, equal amounts (20 µg) of samples were mixed and boiled with SDS loading buffer for 10 min, allowed to cool on ice, loaded into SDS–polyacrylamide gel, and separated by Cleaver electrophoresis unit (Cleaver, UK), transferred onto polyvinylidene fluoride (PVDF) membranes (BioRad) for 30 min using a Semi-dry Electroblotter (Biorad, USA) at 2.5 A and 25 V for 30 min. The membrane was blocked with 5% nonfat dry milk in TBS-T for two hours at RT, to reduce non-specific protein interactions between the membrane and the antibody. The membrane was incubated overnight at 4 °C with primary antibodies against casp-3 (Cell Signaling Technology) and β-actin (Sigma, Aldrich, USA). β-actin is used as a housekeeping protein to enable a more trustworthy interpretation of western blot results. The blots were then washed three times (10 min each) with TBS-T. The membrane was incubated with the corresponding horse radish peroxidase (HRP)-linked secondary antibodies (Dako, USA) for another 60 min at room temperature, washed as previously. The chemiluminescent Western ECL substrate (Perkin Elmer, Waltham, MA) was a 65’8 based imager (Chemi Doc imager, Biorad, USA), and the band’s intensities were measured by ImageLab (Biorad) Protein-sized markers were used in all gels to localize the gel transfer regions for specific proteins and determine the transfer efficiency.

### Statistical analyses

The results were expressed as mean ± standard deviation (SD). The comparison between two groups was assessed statistically using an independent student *t* test. While the comparison among more than two groups was assessed using one-way ANOVA with Tukey’s multiple comparisons post hoc test. Testing for comparison of survival curves was done using Log-rank (Mantel-Cox) test. Tests were analyzed using GraphPad Prism software, version 6. A probability value of less than 0.05 was considered statistically significant.

## Results

### Cytotoxic effect using MTT assay

To evaluate the cytotoxicity effect of the MMR vaccine on EAC cells, the in vitro assay was performed using MTT cell-based assay. Its cytotoxic effect on EAC cells showed that the viability was vaccine concentration-dependent, where the viability increased as the concentration of the vaccine decreased. The IC-50 value of the vaccine was 238 ± 18CCID50/dose.

### Solid tumor growth and EAC‑bearing mice survival rate

**Fig. 1:**
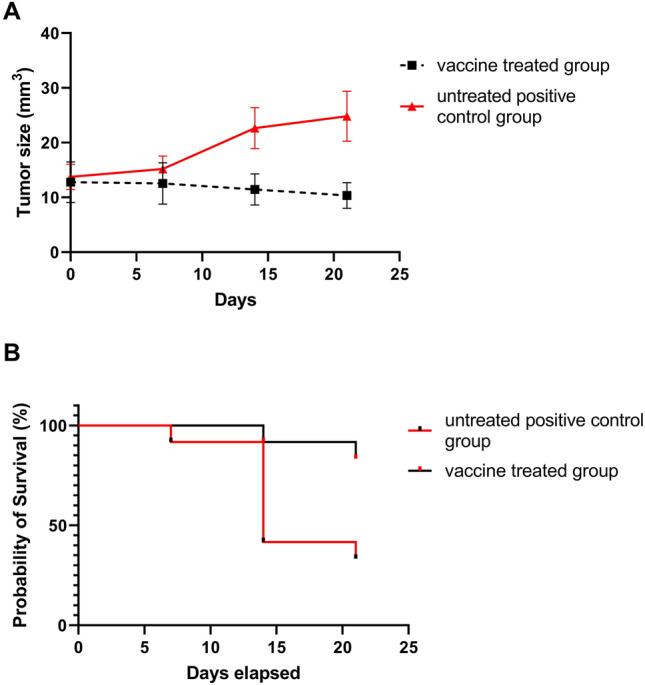
Effect of MMR vaccine on the survival and the solid tumor growth of EAC‑bearing mice. Mice bearing Ehrlich ascites carcinoma (EAC) tumor were injected intratumorally with either MMR vaccine or saline twice weekly. The tumor growth and survival rate were recorded after injection. **A** The size of the tumor in the two groups; **B** survival percentage of tumorized mice groups. Data are expressed by mean ± SD

Mice bearing Ehrlich ascites carcinoma (EAC) tumor were injected intratumorally with either MMR vaccine or saline twice weekly. The tumor growth and survival rate were recorded. The group of EAC-bearing mice treated with MMR vaccine significantly reduced the tumor size compared to the positive control group (*P* value < 0.01). Moreover, the survival rate in the treated group was about 83% which is significantly higher than the controlled group 33%. The results were illustrated in Fig. 1.

### Biochemical analysis

The results of biochemical parameters of serum and tissue levels in all mice groups whether in EAC‑bearing mice or negative control group were summarized in Table 1. As expected, the tumor had a significant intoxication effect as observed in elevated levels of LDH, MDA, NO, GOT and GPT in the EAC‑bearing positive group compared to normal levels in the negative controlled group. Furthermore, the antioxidant status was significantly reduced in the positive group as observed in the lower level of both GSH and PON-1 compared to the negative group. These negative impacts were significantly improved in the MMR-treated EAC‑bearing mice. The levels of liver enzymes especially LDH significantly reduced in vaccine-treated group compared to the untreated one. Also, the same effect was observed in the levels of the oxidation markers (MDA and NO). Whereas, the levels of antioxidants for both GSH and PON-1 were elevated in the treated group than that detected in untreated bearing mice, but still less than the levels in the negative control group.

**Table 1:** Biochemical parameters in serum and tissues of EAC-bearing mice group

Groups/parameters	LDH (U/l)	GOT (U/l)	GPT (U/l)	MDA (nmol/g)	NO (umol/g)	PON-1 (kU/l)	GSH (umol/g)
Negative control group	890.5^p^ ± 218.3	81.72^p,t^ ± 10.6	30.98^p,t^ ± 4.3	96.9^p,t^ ± 6.9	17.4^p,t^ ± 1.2	91.2 ± 9.6	34.5^p,t^ ± 3.9
Positive untreated group 1	2936^n^^,t^ ± 303.3	304^n^^,t^ ± 46.4	69.04^n,t^ ± 7.8	227.3^n,t^ ± 6.7	42.6^n,t^ ± 4.4	58.4^n^ ± 18.3	14.7^n,t^ ± 0.9
Treated group 2	619.9^p^ ± 64.73	177.5^n,p^ ± 15.73	51.9^n,p^ ± 5.2	172.73^n,p^ ± 26.2	31.8^n,p^ ± 6.9	74.25 ± 10.5	27.5^n,p^ ± 2.9

^n^Significant difference compared to the negative control group

^p^Significant difference compared to the positive untreated group

^t^Significant difference compared to treated group

### Histopathological study

**Fig. 2:**
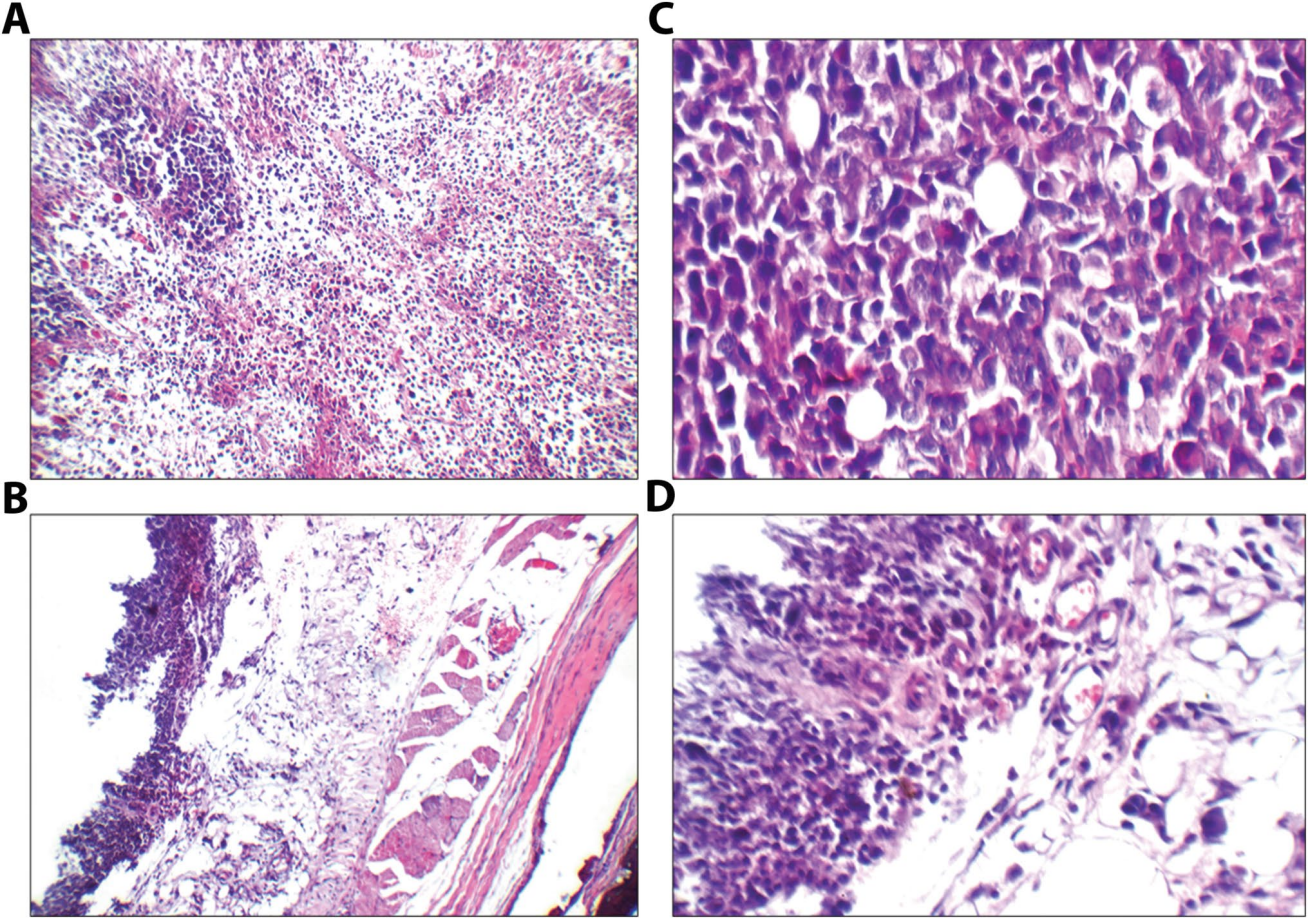
Histopathological demonstration of Ehrlich solid tumor-bearing mice either treated with MMR vaccine or untreated stained with hematoxylin and eosin (H & E). **A** section of tumor in untreated mice group infiltrating tumor composed of large nodules of viable markedly pleomorphic tumor cells (black arrow) with scattered apoptosis (red arrow), and small areas of necrosis (blue arrow) (×200); **B** another view showing infiltrating tumor composed of large nodules of more viable markedly pleomorphic tumor cells (black arrow) with scattered apoptosis (red arrow) infiltrating subcutis (yellow arrow) (×400); **C** section of tumor in MMR vaccine-treated mice group showing high power view showing viable skeletal muscles (black arrow), free bone tissue (yellow arrow), and viable subcutis (blue arrow) with small areas of viable tumor tissue (red arrow) (×200); **D** higher power view showing viable subcutis (black arrow) with small areas of viable mildly pleomorphic tumor cells (yellow arrow) with marked apoptosis (blue arrow), and mildly congested blood vessels (red arrow) (×400)

The tumor section in the positive EAC group showed infiltrating tumor composed of sheets and large nodules of more viable markedly pleomorphic tumor cells infiltrating subcutis with scattered apoptosis, scattered mitosis, and small areas of necrosis. While, the tumor section in the treated group showed subcutis with small areas of viable tumor tissue composed of mildly pleomorphic cells, marked apoptosis as shown in Fig. 2A–D.

### Immunohistochemistry analysis

**Fig. 3:**
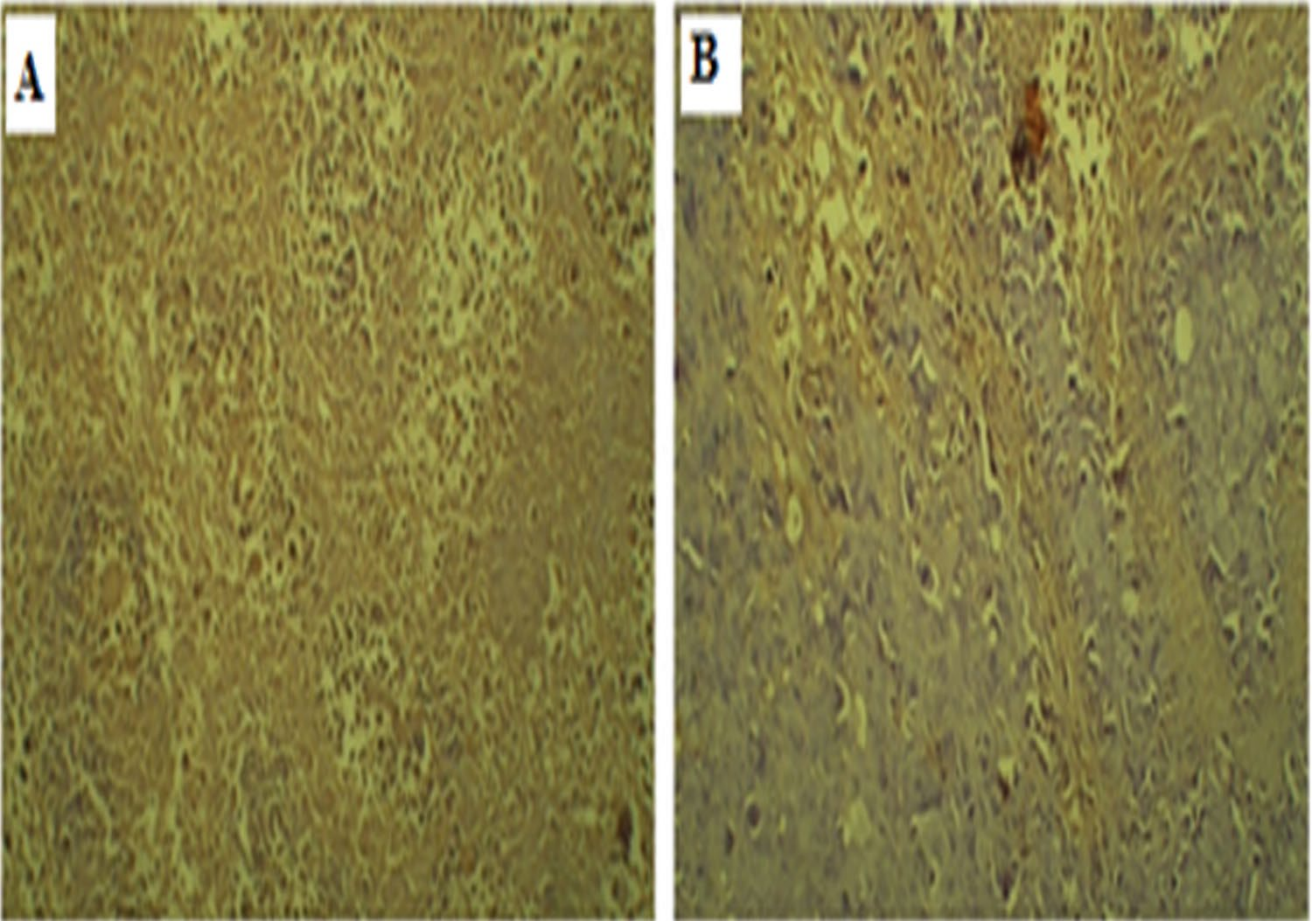
PCNA immunoreactivity of Ehrlich solid tumor-bearing mice treated with MMR vaccine against untreated positive control. **A** Extensive brown spots from DAB staining indicate strong and diffuse nuclear immunoreactivity of tumor cells to PCNA in the tumor tissue of the untreated mice group. **B** Focal and mild nuclear immunoreactivity of tumor cells to PCNA in the MMR vaccine-treated tumor tissue

Immuno-histochemistry biomarker (PCNA) was detected in Ehrlich solid tumor-bearing mice either treated with MMR vaccine or untreated. The PCNA immunohistochemical analysis of the EAC tumor tissue showed a noticeable reduction of the immunoreactivity to PCNA in the samples of the treated mice group compared to the positive group as shown in Fig. 3A and B

### Detection of apoptosis marker and cells in EAC‑bearing mice groups

**Fig. 4:**
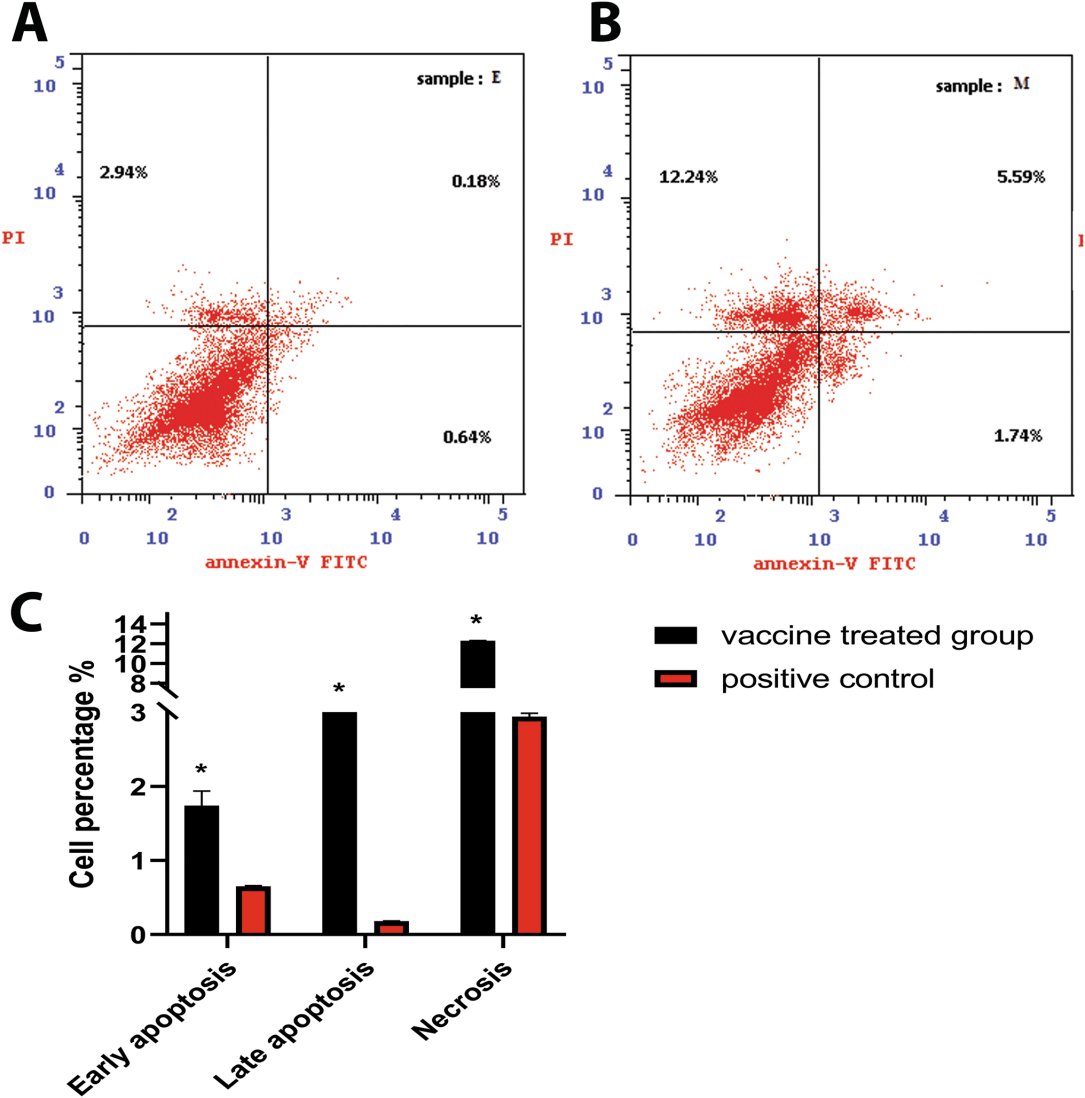
Apoptosis induction by MMR vaccine on the solid tumor of EAC‑bearing mice Ehrlich ascites carcinoma (EAC) cells harvested from mice tumors after 21 days of tumor inoculation were analyzed by flow cytometry in figures **A** and **B**. Flow cytometry dot plot: horizontal axis represent Annexin V binding and vertical axis represent PI uptake. The section on the lower left side represented live cells where FITC-labeled Annexin V and PI both had low fluorescence values. The lower right side represented early apoptosis where FITC-labeled Annexin V had a high fluorescence value, PI was low, the higher right side represented late apoptosis where FITC-labeled Annexin V and PI both had high fluorescence values; while the higher left side represented necrosis cells where FITC-labeled Annexin V had low fluorescence value, PI was high. **A** untreated positive EAC‑bearing mice group **B** tumorized mice group treated with MMR vaccine. **C** bar chart represents the mean of cells % ± SD in each group. *represents significance against the positive group

The capabilities of the MMR vaccine to induce apoptosis or necrosis in tumor tissue were further examined by detecting the apoptosis and necrosis of tumor cell suspension using a flow cytometer. Recorded data revealed that the total apoptotic and necrotic cells in MMR-treated tumor cells was 19.63% ± 0.07, while in untreated tumor cells were 3.54% ± 0.06 (*P* value < 0.001) as represented in Fig. 4A and B

### Immunochemical assay

**Fig. 5:**
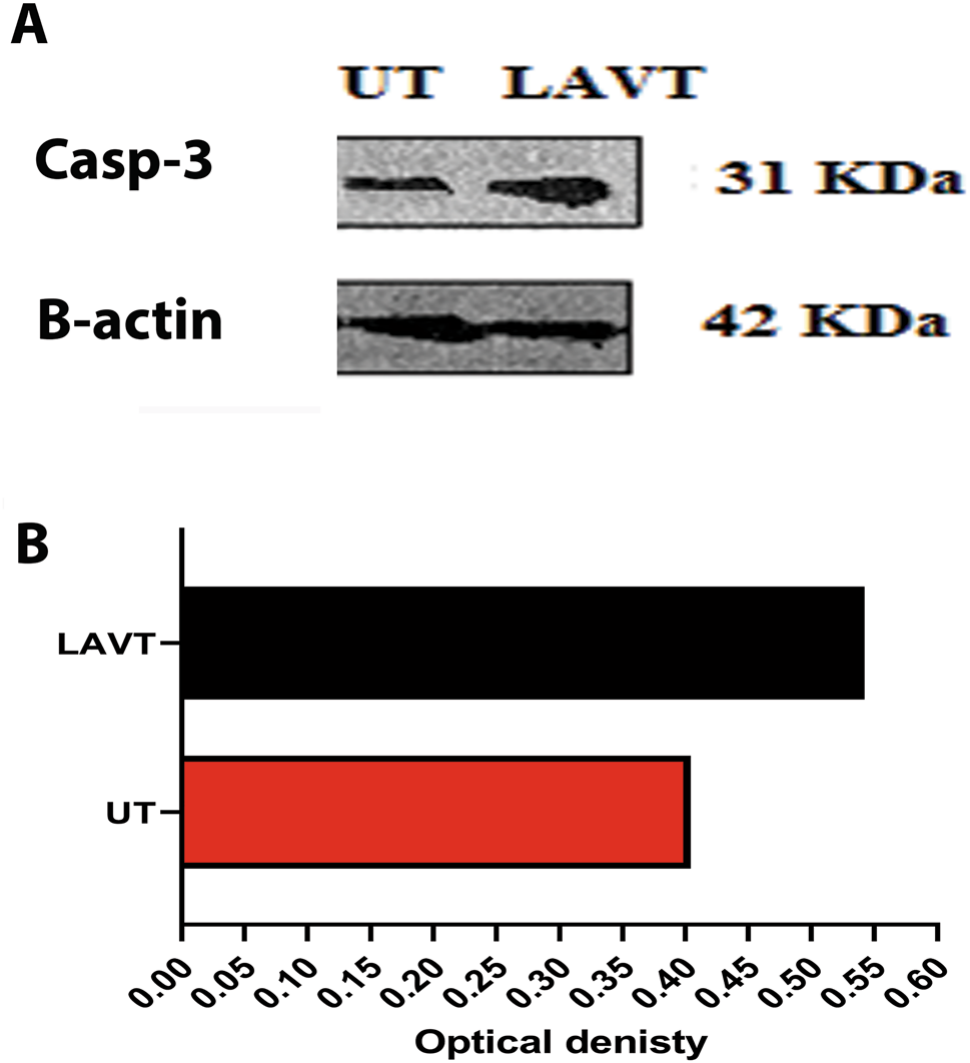
Caspase-3 (Casp-3) induction by MMR vaccine on the solid tumor of EAC‑bearing mice. Casp-3 was examined by western analysis in the harvested tumor in both groups using β-actin as a loading control. **A** The membrane section where UT and LAVT represented untreated positive EAC‑bearing mice and the treated group, respectively. **B** Bar chart where each bar represents the absorbance values of the group optical density

The mode of action of the MMR vaccine on the solid tumor was further investigated using western analysis to detect apoptotic caasp-3 protein in harvested tumor tissues. It was found that a noticeable increase in casp-3 in tumor tissues of vaccine-treated group than in the positive group as shown in Fig. 5A and B.

## Discussion

Oncolytic viruses (OVs) are gaining importance as therapeutic agents for cancer. They provide the appealing therapeutic combination of immune activation and tumor-specific cell lysis which may serve as anticipated in situ tumor therapy [4–11, 24–26]. MMR vaccine is a licensed vaccine worldwide that has already well-established safety and efficacy profile. In the present study, we evaluated the antitumor activities of the WHO-prequalified MMR vaccine from SII by measuring its ability to inhibit tumor growth and enhance the body bio-functions in EAC-bearing mice. First, we examined its in vitro cytotoxicity effect; our results showed that a fifth of the MMR vaccine dose caused a half-inhibitory effect (IC_50_) on the EAC cancerous cells in two days. To demonstrate the vaccine antitumor effect in vivo, the same dose was administrated twice weekly in induced solid EAC-tumor-bearing mice for 21 days. The first judging criteria was the ability of the vaccine to reduce tumor growth and extend the survival rate in tumor-bearing mice. As expected, the vaccine-treated group showed a significant reduction in the tumor size thereby the death rate in the treated group was 16% compared to the untreated group 67% which indicated that the vaccine remarkably prolonged the life span in the treated group. The solid tumor reduction may be attributed to the inhibition effect of the vaccine on the EAC cells proliferation, enhancement of apoptotic pathways against cancerous cells, or the vaccine’s direct necrotizing effect as shown in the in vitro assay.

Further confirmation of our findings came from histological analysis of the tumor mass, which revealed a decrease in tumor infiltration and a rise of apoptotic bodies in the vaccine-treated group. Moreover, we evaluated the proliferation of the tumor cells using PCNA as an important marker of proliferation, which has an important role in triggering the start of the cell cycle and boosting the G1-S phases of the cell cycle [27]. The expression of PCNA in the tumors of vaccine-treated group was noticeably reduced compared with the untreated group.

To assess the apoptotic and necrotic effects of the vaccine, the percentage of apoptotic and necrosis cells were evaluated using flow cytometry. The apoptotic and necrotic cells in the vaccine-treated tumor were 7.33% and 12.24%, while in the control group were 0.82% and 2.94%, respectively. Caspase-3 is one of the important apoptosis regulators activation of caspases-8 leads to DNA damage and subsequent cell death [28–30]. Our results revealed that there was a rise in the expression of Casp-3 in the vaccine-treated tumor compared to the untreated one which confirmed the vaccine attribution to the defective apoptosis enhancement.

To evaluate the effect of the vaccine in restoring the deterioration in the biochemical parameters of serum and tissue levels in tumorized mice, these parameters were assessed in all mice including the untumorized group (negative mice group). The liver enzymes of LDH, GOT and GPT in the blood sera of the untreated EAC-bearing mice showed substantial elevation in these enzymes’ levels related to their level in the normal sera of the negative group. The highest level was observed in the serum LDH which resulted from the dissipation of the dividing malignant cell membrane whose metabolic characteristic is anaerobic glycolysis which causes a rise in LDH enzyme activity as shown in the previous studies [31–33]. Treating the EAC-bearing mice with MMR vaccine significantly diminished the elevated levels of all the mentioned parameters indicating the vaccine’s protective action on body functions.

The oxidation stress markers as MDA and NO were evaluated in body tissues of all mice groups. The rise in MDA levels in the EAC-bearing mice compared to the normal levels in the negative control group could be due to the release of the byproduct of lipid peroxidation during the oxidative breakdown of malignant tissues [34]. On the other hand, Nitric Oxide (NO) regulates the rate of cancer progression and has distinct roles in tumor cells [35]. Our findings exhibited a significant reduction in both MDA and NO levels in the vaccine-treated group toward values of the normal control group. These findings were observed also in the levels of the antioxidant markers of GSH and PON-1. GSH is a vital antioxidant that is capable of inhibiting the tumor formation process, while PON-1 has its antioxidative and anti-inflammatory activities [36, 37]. Therefore, the presence of tumors reduced their levels significantly in the untreated tumor group. Collectively, the results showed that the MMR vaccine was associated with the diminution of cellular damage by suppressing the formation of free radical production and amelioration of the cellular antioxidant defense in tumor-bearing mice.

Our findings complied with the studies that described the antitumor activities of these three attenuated viruses in the MMR vaccine; the first is the Measles virus which already showed its abilities to selectively inhibit the proliferation of tumor cells and enhance the apoptosis defense against them as described in previous studies [38, 39]; the second Mumps virus which caused the death of cancer cells [40]; finally Rubella virus which induced the apoptosis mechanism [41].

In summary, the antitumor activity of MMR prequalified vaccine was assessed in EAC-bearing mice. It was found that the vaccine significantly reduced the proliferation of solid tumor proliferation, and extended the life span of the tumorized mice. The oncolytic effect of the vaccine included activation of the apoptosis pathway as well. Moreover, the vaccine helped in restoring the biochemical parameters in serum and tissues of tumor-bearing nice toward the normal value. Additionally, the MMR vaccine significantly reduced tumor-induced oxidative stress through a variety of mechanisms that included modulating lipid peroxidation and enhancing endogenous antioxidant status. Addition investigation should be done to adjust the use of this vaccine as a curative vaccine either alone or combined with other anticancer therapeutics for different cancer types.

## Data Availability

The datasets generated during and/or analyzed during the current study are available from the corresponding author on reasonable request.
